# Vestibular stimulation and space-time interaction affect the perception of time during whole-body rotations

**DOI:** 10.1371/journal.pone.0313219

**Published:** 2025-01-15

**Authors:** Deborah Cecilia Navarro Morales, Alexis Laplanche, Olga Kuldavletova, Bithja Cantave, Adéla Kola, Thomas Fréret, Gaëlle Quarck, Gilles Clément, Pierre Denise

**Affiliations:** 1 COMETE U1075, Inserm, CYCERON, Université de Caen Normandie, Caen, France; 2 Department of Clinical Physiology, CHU de Caen, Caen, France; Universidad de Guadalajara, MEXICO

## Abstract

Among the factors, such as emotions, that distort time perception, vestibular stimulation causes a contraction in subjective time. Unlike emotions, the intensity of vestibular stimulation can be easily and precisely modified, making it possible to study the quantitative relationship between stimulation and its effect on time perception. We hypothesized that the contraction of subjective time would increase with the vestibular stimulation magnitude. In the first experiment, participants sat on a rotatory chair and reproduced time intervals between the start and the end of whole-body passive rotations (40° or 80°; dynamic condition) or between two consecutive low-amplitude shakes (static condition). We also assessed reaction time under the same conditions to evaluate the attentional effect of the stimuli. As expected, duration reproduction in the 40° rotation was shorter than that observed in the static condition, but this effect was partly reversed for 80° rotations. In other words, vestibular stimulation shortens the perceived time interval, but this effect weakens with stronger stimulation. Attentional changes do not explain this unexpected result, as reaction time did not change between conditions. We hypothesized that the space-time interaction (i.e., spatially larger stimuli are perceived as lasting longer) could explain these findings. To assess this, in a second experiment participants were subjected to the same protocol but with three rotation amplitudes (30°, 60°, and 120°). The duration reproductions were systematically shorter for the lower amplitudes than for the higher amplitudes; so much so that for the highest amplitude (120°), the duration reproduction increased so that it did not differ from the static condition. Overall, the experiments show that whole-body rotation can contract subjective time, probably at the rather low level of the interval timing network, or dilate it, probably at a higher level via the space-time interaction.

## Introduction

The subjective duration of an event can be influenced by a multitude of factors, such as task complexity [1], familiarity [2], attention and arousal [3], volition [4], and emotions [5], including fear [6]. Time perception distortions are usually interpreted in the frame of the pacemaker-accumulator model [7] and the attentional gate model [8], in which a) pulses, regularly emitted by a pacemaker, are temporarily stored in an accumulator; b) the quantity of pulses transferred from the pacemaker to the accumulator is controlled by an attentional gate; and c) the accumulator sends the total count to working memory, which compares it to past experiences and decisional processes determine the time represented by the total pulses. Empirical findings have shown that arousal and attention are the two main factors affecting temporal processing [3]. An increase in the level of arousal increases the rate of the pacemaker, thus dilating subjective time, whereas when attention is not entirely allocated to the evaluation of time, the attentional gate transfers fewer pulses to the accumulator, thus contracting subjective time. Recently, it has been shown that stimulation of the vestibular system could also distort time perception. When subjected to passive vestibular stimulation induced by the oscillatory movements of a swing, participants consistently produced longer durations than at rest [9], suggesting that vestibular stimulation contracts subjective time. Within the pacemaker-accumulator model framework, the overproduction of time intervals during vestibular stimulation can be explained either by a pacemaker slowing down or by a reduction in the number of impulses transferred by the attentional gate to the accumulator. Consequently, in both cases, the accumulator would take longer to accumulate the same number of impulses during swinging than at rest. Based on the results of two other experiments carried out as part of the same study, Utegaliyev et al.(2022) [9] hypothesized that the effect of vestibular stimulation on the timing network is not mediated by a change in arousal or attention, but directly by an increase in the number of accumulator misses. However, it is known that attention is automatically captured by stimuli moving towards the observer [10] especially if they are on a collision path [11]. It is reasonable to think that the same effect should be observed in the symmetrical situation (*i*.*e*., when the observer is moving towards the object) and therefore during passive rotation where the risk of collision with surrounding objects is greater. We can therefore hypothesize that the contraction of subjective time observed during vestibular stimulation is mediated by attentional processes.

Unlike most other factors that distort the perception of time, the intensity of vestibular stimulation can be very easily and precisely modified. Vestibular stimulation can be multiplied by an arbitrary factor, whereas this is not possible for an emotion. If the vestibular system acts directly on the timing network, it becomes possible to study the quantitative relationship between stimulation and its effect on the perception of time. This study aimed to assess the effect on time perception and attention of vestibular stimulations of different magnitudes. We hypothesize that the contraction of subjective time will increase with the magnitude of the vestibular stimulation.

## Experiment 1: Time estimation during whole-body rotation

### Methods

#### Participants

Fifteen healthy participants (12 women, 3 men; mean age M+/-SD: 22.3+/-1.87) voluntarily participated in Experiment 1. None of the participants had a history of vestibular, neurological, or auditive disorders. The study adhered to the ethical standards of the Declaration of Helsinki and was approved by the research ethics committee ‘*Comité de Protection des Personnes de la Région Ouest’* (ID-RCB 2022-AO1513-40). Participants constituted the control group of a study on bilateral vestibulopathy and were recruited from 3 October 2022 to 18 January 2024 and provided written informed consent.

#### Experimental procedure

**Fig 1:**
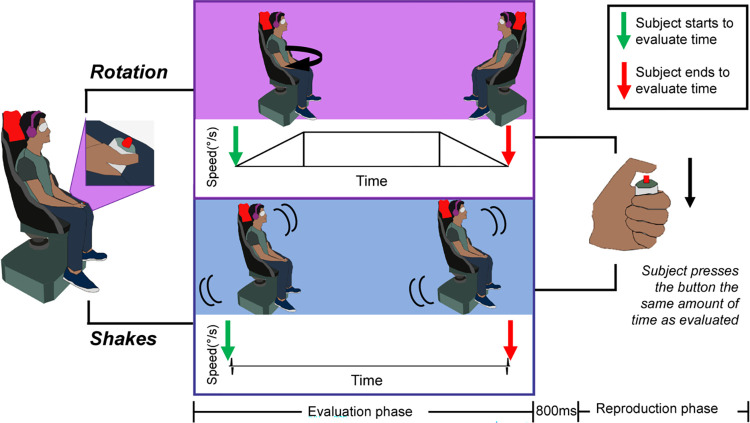
Experimental protocol and method. From left to right: The participant sat on a rotatory chair, he/she was equipped with an active anti-noise headset, a mask, and held a response button in his/her preferred hand. During the evaluation phase, the rotatory chair could rotate (upper panel) or deliver two low-amplitude shakes (static condition) separated by a time interval (lower panel). The participant had to evaluate the total time of the action (from green arrow to red arrow). In the reproduction phase, the participant pressed the button for the same amount of time as previously evaluated.

The participants sat on a rotatory chair and were equipped with an active anti-noise headset (Sennheiser® HD 450BT), a facial mask, and a response button in their preferred hand. Each trial was performed in darkness and composed of an evaluation phase and a reproduction phase. In the evaluation phase, the rotatory chair could rotate (dynamic condition) or deliver two consecutive low-amplitude shakes (static condition) (Fig 1).

In the dynamic condition, the rotating chair turned 40° or 80° to the right or left within 2 or 4 seconds. Each rotation started and ended with a 1-second acceleration/deceleration period separated, for a 4-second stimulation, by a 2-second plateau of constant velocity. Consequently, for a given duration, the stimulation profile was the same for the 40° and 80° trials, but with an amplitude and velocity, and therefore a magnitude of vestibular stimulation, twice as great for the 80° trials.

In the static condition, two shakes of the chair were separated by an interval of time during which the chair was stationary. The shakes were very brief and low amplitude, each consisting of two consecutive rotations in opposite directions with an amplitude of 1° and a speed 80°/s; the vestibular stimulation was, therefore, very low. The total duration (the shakes + the interval of time between shakes) was 2 or 4 seconds.

Participants were instructed to evaluate the time elapsed between the start and end of the rotation or, in the case of shakes, between the start of the first shake and the end of the second shake. No specific time evaluation strategy was imposed (counting was not forbidden) but the participants had to keep the same strategy throughout the test. After each stimulation, there was a pause of 800 ms, followed by a vibration of 200 ms applied to the response button to announce that participants had to begin time reproduction. The participants pressed the button for the same length of time as previously evaluated. Finally, 5 seconds of rest were applied before the next trial to avoid post-rotational effects. Each participant completed 4 training trials to get familiarized with the test, followed by 48 experimental trials presented in a counterbalanced order. The 48 experimental trials consisted of 8 repetitions of each combination of rotation conditions (static, 40°, 80°) and time intervals (2 or 4s).

The attentional effect of the stimulation was evaluated with a reaction time task. The participants were installed in the same way as for the reproduction of the duration task. The rotatory chair, as previously, could rotate or deliver shakes, and 5 seconds of rest were given between trials. Each trial lasted 2 or 4 seconds. Vibrations to the response button (200 ms duration) were randomly delivered during rest or after the start of rotations or shakes (500, 750, 1000, 1250, 2000, or 3000 ms) and the participants had to react as quickly as possible by pressing the button. Participants were instructed to pay attention only to the button vibration and not to the rotatory chair stimuli. The participants did 4 trials for training and then 42 experimental trials.

#### Statistical analysis

In the duration reproduction task, the mean of the perceived duration per condition was computed for each participant. Then we calculated the mean relative estimation error, which is given by [9]

$$\frac{{interval}_{reproduced}-{interval}_{to-be-reproduced}}{{interval}_{to-be-reproduced}}.100[\%]$$

Then, the relative estimation error was analyzed with a two-way Repeated Measures ANOVA (two-tail), with the real stimulus duration (2 and 4 seconds) and the stimulus type (static, 40 or 80 degrees) as the fixed effect. A Greenhouse-Geisser correction was applied when the sphericity assumption was not met. Then, a *post-hoc* test was conducted with Holm correction.

Reaction time was analyzed using a one-way Repeated Measures ANOVA (two-tail), with the stimulus type (static, 40 or 80 degrees) as the fixed effects and reaction time as the dependent variable. The significance level (α) was set at 0.05. All tests were performed using the JASP 0.17.2.1. statistical software. Individual differences that could constitute confounding factors, such as vestibular sensitivity, cognitive factors, or prior experience of similar tasks are taken into account by the repeated measures models we used.

### Results

Table 1 presents an overview of the duration reproductions during the different stimuli of the rotatory chair.

**Table 1:** Results (seconds) and descriptive statistics of the Experiment 1.

Condition	2 seconds			4 seconds		
	*M ± SD*	CI 95%		*M ± SD*	CI 95%	
		*lower*	*upper*		*lower*	*upper*
0° (Static)	1.717 ± 0.550	1.439	1.995	3.266 ± 0.604	2.960	3.571
40°	1.451 ± 0.451	1.223	1.680	2.865 ± 0.585	2.569	3.161
80°	1.550 ± 0.444	1.325	1.775	3.112 ± 0.558	2.830	3.395

**Fig 2:**
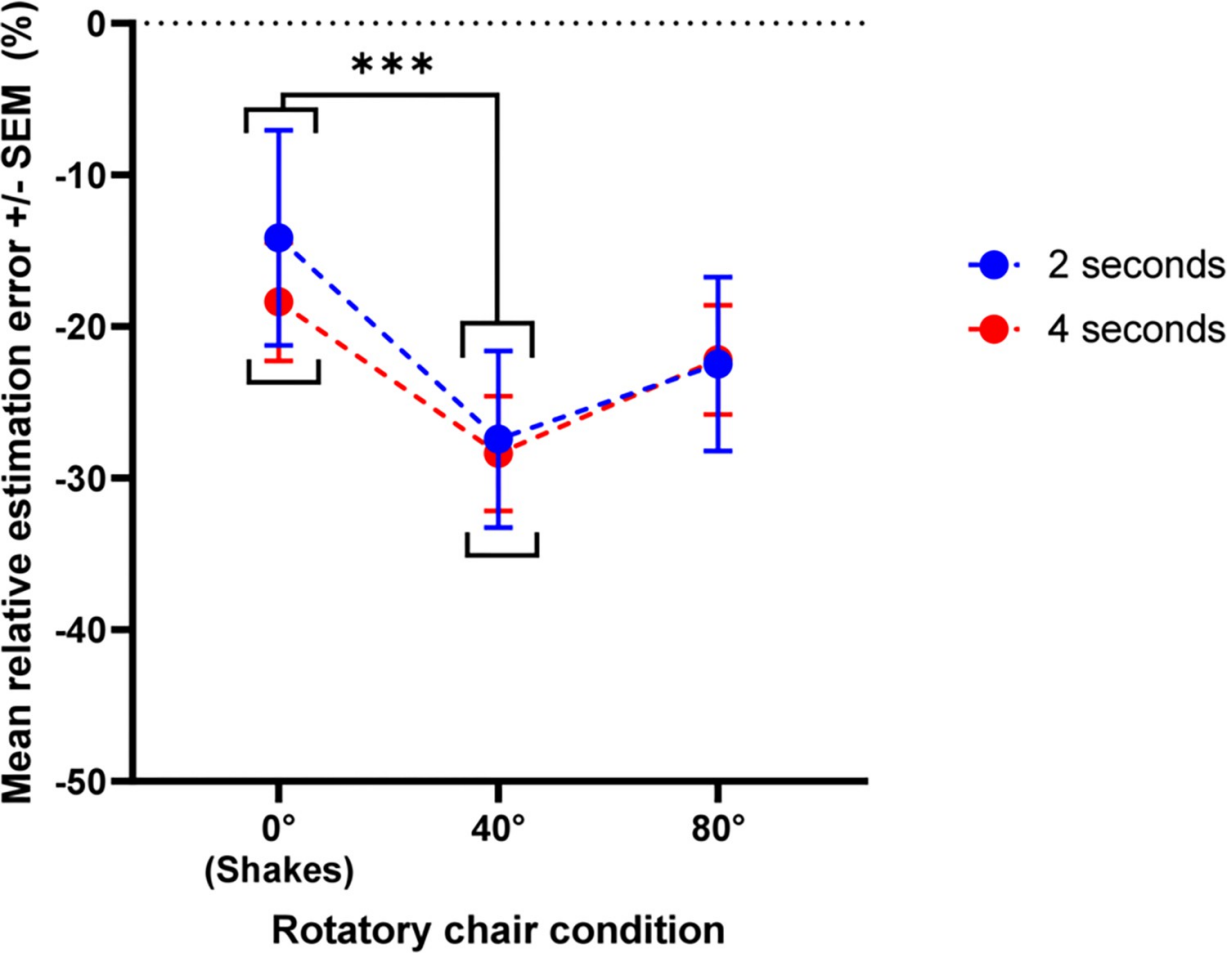
Mean relative estimation error for each for each to-be-reproduced duration and type of stimulation (Experiment 1). Error bars represent +/- 1 standard deviation of the mean.

Fig 2 shows the mean relative estimation error as a function of stimulus type (static, 40°rotation, 80° rotation) and duration (2 or 4 seconds). There was no significant interaction between the type and duration of the stimulus *[F (2*,*28) = 0*.*725*, *p = 0*.*493*, *η2 = 0*.*008]*. The mean relative error varied significantly depending on the type of stimulus *[F (1*.*080*, *15*.*124) = 9*.*225*, *p = 0*.*007*, *η2 = 0*.*195]*.

The duration reproductions were longer for the static condition than for the 40° condition *(p<0*.*001)*. However, there were no statistical differences when comparing the static condition to 80° rotations (p = 0.066), although there was a trend toward significance. Similarly, no statistical difference was observed comparing 40° to 80° rotations (p = 0.066), but again, there was a trend to significance. The duration of the stimulus (2 or 4 seconds) did not affect the duration reproductions *[F (1*,*14) = 0*.*229*, *p = 0*.*640*, *η2 = 0*.*006]*.

**Fig 3:**
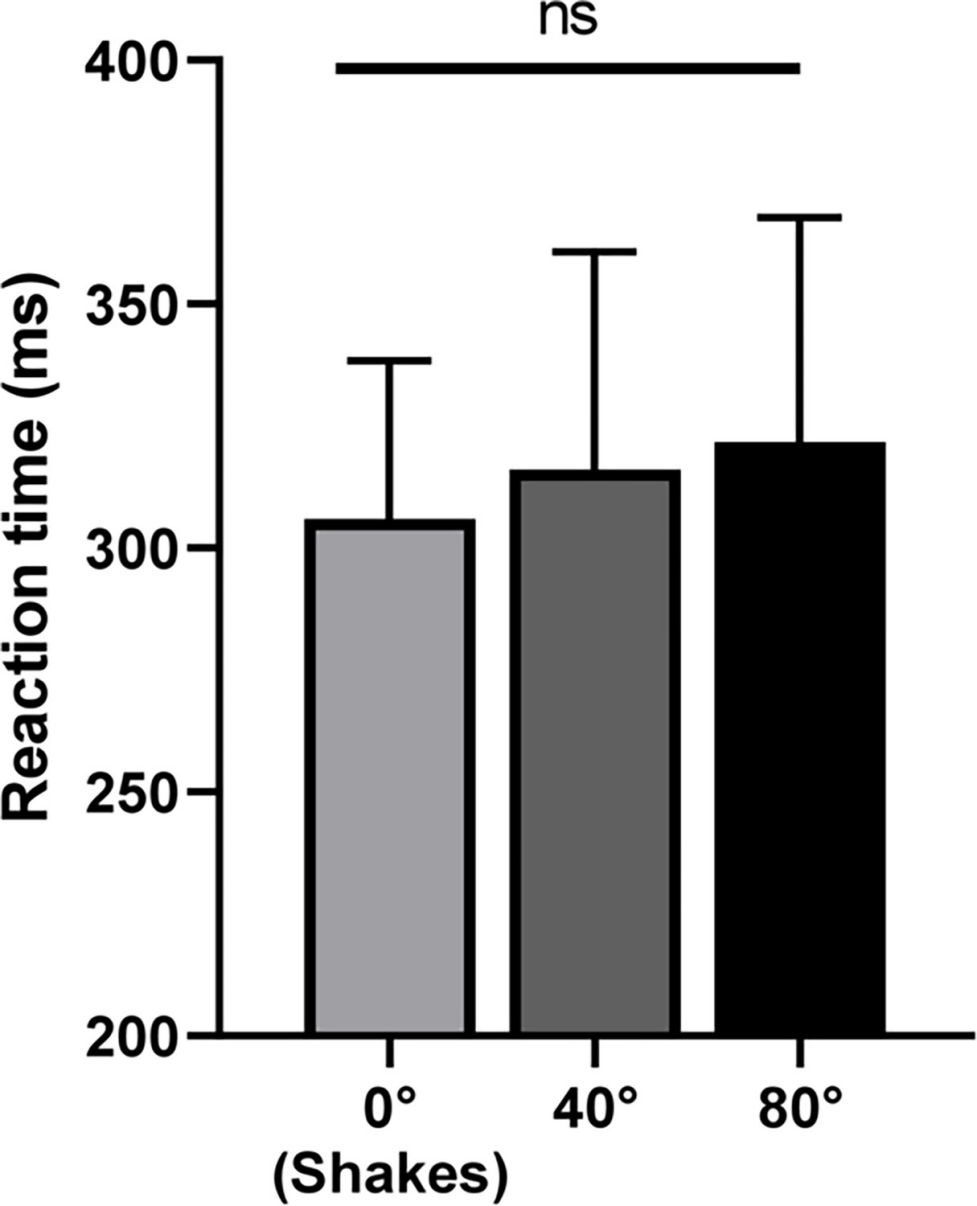
Reaction time task for each type of stimulation (static, 40° rotation, and 80° rotation). Error bars represent the standard deviation.

Fig 3 shows the mean reaction time as a function of stimulus type (static, 40° rotation, 80° rotation). There was no effect of stimulus type on reaction time *[F (2*,*26) = 1*.*885*, *p = 0*.*172*, *η2 = 0*.*006]*.

## Discussion

Our results confirm those of Utegaliyev et al. [9] by showing that perceived durations are shorter during whole-body rotations than at rest. Even if our experiment stimulates the vestibular system as in [9], the sensory receptors involved are not the same: In our experiment, rotation of the whole body along a vertical axis in the dark simulates only the lateral semi-circular canals of the vestibular system; in contrast, in [9] rotation around a horizontal axis stimulates both the vertical semicircular canals, the otoliths, as well as the somatic graviceptors [12]. We can conclude from our study that stimulation of the semicircular canals is sufficient to distort the perception of time. It remains to be determined whether isolated stimulation of the otolith organs, for example by linear movement on a sled, also affects the perception of time.

Within the pacemaker-accumulator model framework, stimulation of the semicircular canals would result in fewer pulses being sent to the accumulator, which would therefore count fewer pulses during rotation than during the static condition. During the reproduction phase, the chair did not move anymore, thus, the pulse rate would return to the baseline and it would take less time to accumulate the same number of pulses, leading to under-reproductions in the dynamic rotation conditions.

By showing that reaction time was not modified by vestibular stimulation, our results also confirm the hypothesis of Utegaliyev et al. (2022) [9] that the effect of vestibular stimulation on time perception is not mediated by an attentional effect, a factor known to affect time estimation [8]. Moreover, it is also unlikely that arousal, a factor known to increase the rate of pacemaker [8], was involved because it is known that passive rotation of the head does not induce any change in heart rate, blood pressure, and sympathetic activity [13].

On the other hand, contrary to our initial hypothesis, increasing the amplitude of the vestibular stimulation not only did not increase its effect on the perception of time but partially reversed it, i.e., the perceived duration of an 80° rotation was longer than that of a 40° rotation, even though this perceived duration remained shorter than at rest.

There is therefore an apparent contradiction: the presence of vestibular stimulation shortens the perceived time interval, but stronger vestibular stimulation does not increase this effect but partially cancels it out. This unexpected result cannot be explained by attentional changes, as there were no changes in reaction time between conditions.

When assessing the duration of a movement, the well-known cross-dimensional interference between time and distance could come into play. Cross-dimensional interference is the phenomenon by which magnitudes along different dimensions (e.g., space and time) tend to interact with each other in perception [14]. For instance, Xuan et al. [15] showed that when 2 squares of different sizes were presented on a screen for the same duration, the large square was perceived to appear for a longer duration than the small square. Thus, in our study, when estimating the duration (time dimension) of a self-rotation of increasing amplitude (space dimension), two phenomena could have acted in opposite directions: the vestibular stimulation would cause a contraction of subjective time, while the space-time interference would dilate it. To test this hypothesis, we repeated Experiment 1 with three rotation amplitudes.

## Experiment 2: Time-space interference

### Methods

#### Participants

Thirteen healthy participants (10 women, 3 men; mean age M+/-SD: 22.3+/-1.75) participated in Experiment 2. They were also drawn from the same control group as experiment 1 and selected under the same conditions, including providing written informed consent.

#### Experimental procedure

The experimental procedure was the same as in Experiment 1, except that three rotation amplitudes (30, 60, and 120°) were used instead of two.

#### Results and discussion

Table 2 presents an overview of the duration reproductions during Experiment 2.

**Table 2:** Results (seconds) and descriptive statistics of the Experiment 2.

Condition	2 seconds			4 seconds		
** **	*M ± SD*	CI 95%		*M ± SD*	CI 95%	
		*lower*	*upper*		*lower*	*upper*
0° (Static)	1.657 ± 0.540	1.363	1.950	2.978 ± 0.727	2.583	3.374
30°	1.380 ± 0.387	1.161	1.599	2.431 ± 0.592	2.109	2.752
60°	1.479 ± 0.388	1.268	1.690	2.786 ± 0.525	2.501	3.071
120°	1.570 ± 0.439	1.332	1.809	2.936 ± 0.583	2.619	3.253

**Fig 4:**
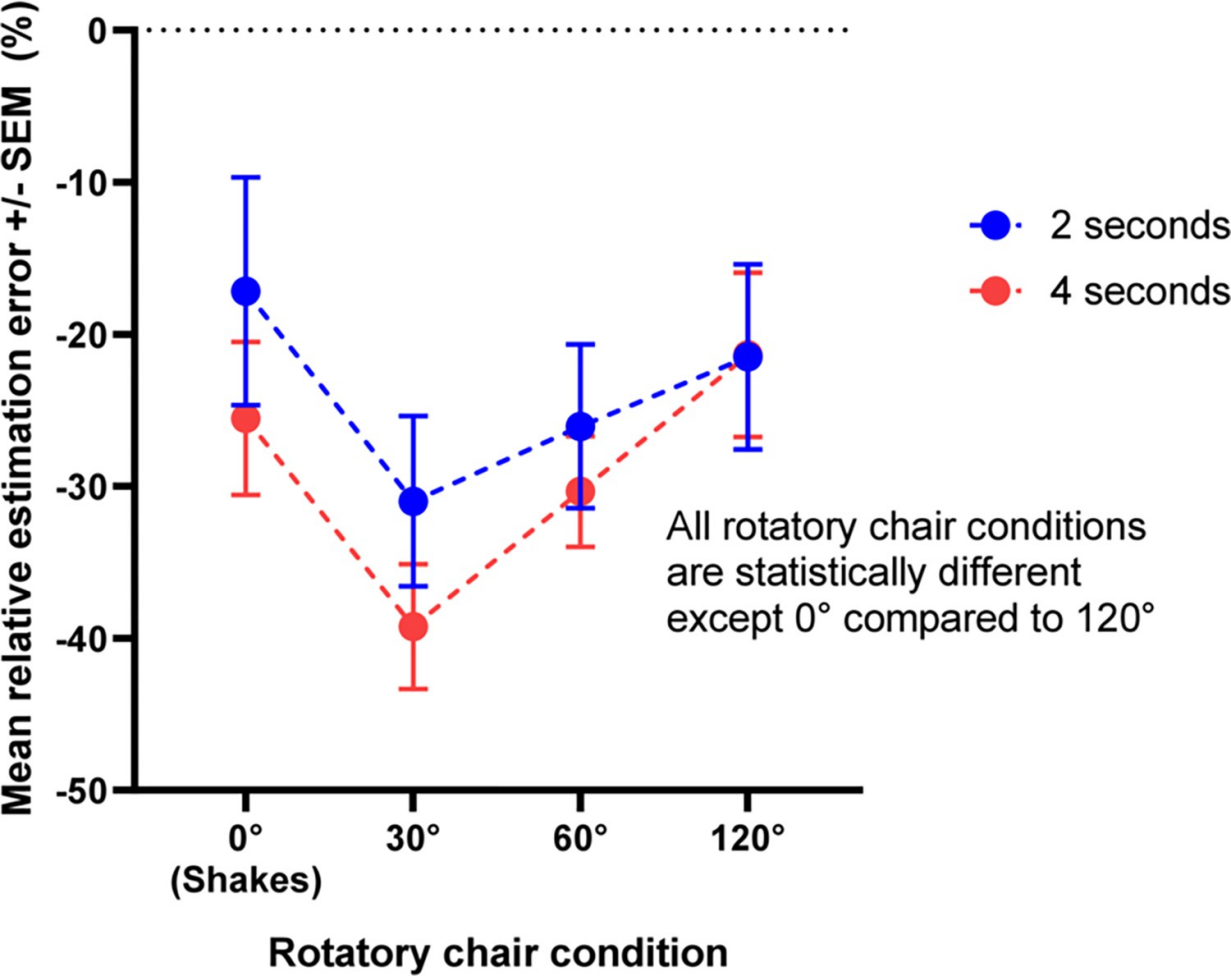
Mean relative estimation error for each for each to-be-reproduced duration and type of stimulation (Experiment 1). Error bars represent +/- 1 standard deviation of the mean.

Fig 4 shows the mean relative error as a function of stimulus type (static, 30° rotation, 60° rotation, 120° rotation) and duration (2 or 4 seconds). There was no significant interaction between the type and the duration of the stimulus *[F (1*.*528*, *16*.*807) = 0*.*983*, *p = 0*.*373*, *η2 = 0*.*021]*. The mean relative error varied significantly depending on the type of stimulus *[F (1*.*648*,*18*.*129) = 12*.*293*, *p<0*.*001*, *η2 = 0*.*207]*. Duration reproductions in the static condition were longer than those for 30*° (p <0*.*001)* and 60*° (p = 0*.*035)* rotations but did not differ from those for 120° rotations *(p = 0*.*759)*. Furthermore, duration reproductions were shorter during 30° rotation compared to 60° *(p = 0*.*050)* and 120° *(p = 0*.*001)* rotations. Finally, the perceived duration during 60° rotations was shorter than 120*° (p = 0*.*050)*. The duration of the stimuli did not affect the duration reproductions *[F (1*, *11) = 0*.*999*, *p <0*.*339*, *η2 = 0*.*029]*.

The results of Experiment 2 confirm those of Experiment 1 and support the space-time interference hypothesis by showing that the greater the amplitude of rotation, the longer the perceived duration of rotation. For a 120° rotation, the perceived duration no longer differs from the duration perceived in the static condition.

## General discussion

This study measured time perception during whole-body rotation in yaw in different conditions: static (*i*.*e*. time interval delimited by shakes), and 30°, 40°, 60°, 80° and 120° rotations). During the 30°, 40°, 60° and 80° rotations, participants under-reproduced time intervals compared to the static condition. Moreover, time reproduction during small amplitude rotations was shorter than during large amplitude rotations. These results cannot be explained by changes in attention, as there were no changes in reaction time between the different conditions. We postulate that two phenomena occur simultaneously and compensate to a greater or lesser extent depending on the characteristics of the movement: (1) an effect of vestibular stimulation, and more specifically of the lateral semicircular canals (2) and a space (angular)—time interaction.

In both Experiment 1 and Experiment 2, the participants reproduced shorter time intervals during the rotations than during the static condition. Our results are in line with those of Utegaliyev et al. [9] obtained during stimulation on a swing. Besides, our results provide further information that this contraction of subjective time is due, at least in part, to stimulation of the semicircular canals. As there is no change in reaction time, we can therefore deduce that our results do not reflect changes in levels of arousal or attention, but rather that vestibular stimulation directly affects the timing network. Indeed, time perception neuronal networks appear to receive afference from the vestibular system. The insula [16] has been identified as an interoception and body awareness hub playing a role in time perception [17–19]. The hippocampus [20], contains time cells [21] and processes vestibular inputs. The cerebellum [22] and cortico-striatal pathways [23] are key components of time perception circuitry [24] and are both implicated in vestibular processing. Finally, the intraparietal sulcus and the insula have been related to time perception, to space-time interactions [25, 26] and the vestibular system [27].

In addition to the effect of vestibular stimulation on time perception, we also demonstrated space-time interference. This phenomenon was observed in other modalities such as visual [28, 29], auditive [30], tactile [31], or combinations of modalities [32]. According to the ATOM framework (A Theory of Magnitude), time, space, and numerosity are represented by a common magnitude and influence each other equally [33]. In opposition, the Conceptual Metaphor Model posits that time (abstract measure) is affected by space (concrete measure) but not the other way around [28, 34]. Both models have been supported by experimental evidence [15, 28, 34–41], as a result, there is an ongoing debate over which of these models is correct. However, given we studied only the effect of space over time perception both models apply to our results. During whole-body rotations, the perceived time depends on the amplitude of rotation perceived by the vestibular system: rotations with larger amplitudes are perceived to last longer. It is important to note, that in our results, this interference can be linked to either the amplitude or the velocity of the movement because the change in amplitude and velocity was proportional.

The interaction between vestibular stimulation and space-time interference may explain the puzzling results of Choi et al. [42]. In one of the tasks in this study, participants were subjected to two consecutive whole-body rotations in opposite directions and had to decide which rotation lasted longer. Discrimination of duration was very good, except when the duration of rotations varied in the opposite direction to their amplitude (i.e., longer rotations had a lower amplitude), where the precision of discrimination fell considerably. According to our hypothesis, in this condition, the increase in duration is counterbalanced by time-space interference, because the decrease in amplitude contracts subjective time. Using the same experimental paradigm, Kwon et al. [43] found that duration discrimination was globally impaired in patients with unilateral vestibular lesions. It is interesting to note that the precision returned to a value equivalent to that of the controls when the duration and amplitude varied in the same direction. Again, this may be explained by the time-space interference that here would add its effects to the increase in duration.

A similar time-space interaction has already been demonstrated during virtual displacement [44]. There is some evidence that this interference between travel time and traveled distance is mediated by neuronal representations of movement speed in the right intraparietal sulcus and the left middle temporal complex. This interference at a higher level would be a specific case of common cortical metrics evaluating the magnitudes of time, space, and other quantities (ATOM framework) [33].

Our experiment lacks insight into the otolithic system’s impact on time perception. Yet, the otolithic system may be involved, since swinging leads to time interval overproductions [9] and weightlessness (a state in which tonic stimulation of the otolith system disappears) to underproduction of time interval [45, 46]. Further research is needed to explore how time is perceived during self-translations in order to isolate the effect of otolithic system stimulation.

Our results demonstrate the complex nature of the perception of time, its dependence on the vestibular sensory input, and the time-space interaction. Distortions of time perception might not have a significant impact in daily life, but they could become important in situations where the perception of duration and amplitude of movement is crucial, such as in sports, driving, and piloting as well as in space exploration. Understanding these distortions in the perception of time and amplitude of self-motion would improve our capacity to create efficient training for sportsmen, pilots, and astronauts, and improve operational protocols and environment.

## Supporting information

S1 Dataset (XLSX)
